# Outlines of Practical Hygiene

**Published:** 1894-01

**Authors:** 


### BOOK NOTICES.

Outlines of Practical Hygiene ; Adapted to American Conditions. By C. Gilman Currier, M. D., Visiting Physician to New York City Hospital, etc. New York, 1893: E. B. Treat, 5 Cooper Union. One large octavo volume, 468 pages, illustrated. Price, 2.75.

The intelligent and scientific utilization of all known means for prolonging life and preventing diseases has become a speciality in itself.

Recognizing this fact, and in response to a large and growing demand, we call especial attention to the above indicated volume, which upon investigation will be found to embody the results of the most profound research. It is by far the most recent, scientific, agreeably written and practically instructive, of all the existing books on the subject.

Although a practical sanitarian, and expert specialist, the author, before permitting the book to be issued, submitted his manuscript to the careful criticism of experts in the various departments of the great field which the book covers. We accordingly commend it emphatically as a volume fully abreast of the times, in which accepted truths only are admitted and opinions advanced in accord with leading thinkers and workers.

The book embodies much matter based on the most recent American and European literature that no one can afford to dispense with, who cares to keep in touch with the scientific progress of the day.

